# Investigation of Kelussia Odoratissima and Angelica Sinensis Similarities in Zebrafish-based In-vivo Bioactivity Assays and Their Chemical Composition

**DOI:** 10.31661/gmj.v12i.2793

**Published:** 2023-07-30

**Authors:** Mohammad Rezaei, Parisa Fooladi, Mohamad Norani, Alexander Crawford, Shahram Eisa-Beygi, Yaser Tahamtani, Mahdi Ayyari

**Affiliations:** ^1^ Department of Stem Cells and Developmental Biology, Cell Science Research Centre, Royan Institute for Stem Cell Biology and Technology, ACECR, Tehran, Iran; ^2^ Department of Developmental Biology, University of Science and Culture, Tehran, Iran; ^3^ Department of Horticultural Science, Tarbiat Modares University, Tehran, Iran; ^4^ Faculty of Veterinary Medicine, Norwegian University of Life Sciences, Oslo, Norway; ^5^ Department of Radiology, Medical College of Wisconsin, Milwaukee, Wisconsin, USA; ^6^ Reproductive Epidemiology Research Center, Royan Institute for Reproductive Biomedicine, ACECR, Tehran, Iran

**Keywords:** Angiogenesis Inhibitors, Pancreatic Beta Cell, Zebrafish, Essential Oil

## Abstract

Background: Kelussia odoratissima and Angelica sinensis are two medicinal plants commonly used in Iran and China, respectively. They have been used in their indigenous traditional medicine, for various diseases including, blood refining, inflammation, cold, flu, stress, cardiovascular diseases, and nervous disorders. This study was conducted to evaluate the volatile oil composition of K. odoratissima leaves (KVL) and A. sinensis root (AVR); we also examined the biological activity of essential oils (EOs) and hydroalcoholic extracts of both plants using two different transgenic zebrafish (Danio rerio) models: angiogenesis and pancreatic beta cell (pBC) regeneration models.Materials and Methods: Both EOs were isolated by hydrodistillation and analysed by GC and GC/MS. For viability tests, larvae were treated with different concentrations of extracts to determine an appropriate starting concentration. Hydroalcoholic extracts and EOs have been tested in a dose-dependent manner for their biological activity using tissue-specific transgenic zebrafish Tg(fli-1: EGFP) and Tg (ins: GFP-NTR) embryos and larvae. One-way ANOVA was used to compare the mean of pBC area and intersegmental vessels (ISVs) outgrowth between the treatment groups.Results: Eleven compounds were in common to both oils, comprising 51.3% of KVL and 61.7% of AVR, of which 39.3% in KVL and 37.6% in AVR were phthalide structures. Results revealed that both EOs blocked ISVs formation in the Tg (fli-1: EGFP) embryos increased to 10% of the control value, while both hydroalcoholic extracts did not show any anti-angiogenesis effects in these embryos. In addition, AVR has been shown to significantly induce PBC regeneration following ablation in the Tg (ins: GFP-NTR), but its regenerative activity was lower than that of 5′-N-ethylcarboxamidoadenosine (NECA) as a positive control. Taken together, the anti-angiogenesis activity of both EOs could be attributed to the phthalide structures while for the PBC regenerative activity, other compounds including β-Thujaplicinol, exclusively existing in AVR, might be effective.Conclusion: Although the genera, organs, and origin of these plants are different, their similar chemical composition and biological activities make them valuable resources for further investigation in basic medical and pharmaceutical science.

## Introduction

Plants containing molecules with phthalide structures have long been of interest in traditional medicine around the world. Natural phthalides are a relatively small group of compounds that exhibit a wide range of biological activities. They exist in several higher and lower plants and are used in many of the traditional medicinal practices in Asia, Europe, and North America [1]. Kelussia odoratissima and Angelica sinensis are the two most abundant sources of phthalides in Iran and China, respectively. K. odoratissima known as "Kelows" or "Karafse kouhi" grows exclusively in central Zagros Mountain mainly in Chaharmahal & Bakhtiari provinces. This plant is consumed traditionally in yogurt-based products and pickles. It has a pleasant aroma and is known for its warm nature, according to Iranian traditional medicine, especially for feminine disorders [2][3]. Angelica sinensis, also known as "Don qui", is one of the most well-known medicinal plants in traditional Chinese medicine for the invigoration of blood and treatment of female irregular menstruation cycles as well as tonifying and relieving pain [4].

It is also used in the formulation of other Chinese medicinal plants for various treatments [5][6]. A long list of potential biological activities associated with phthalides was recently published by León et al. [1], including analgesic, antihyperglycemic, antithrombotic and antiplatelet, neurological effects on Alzheimer’s disease, Parkinson’s disease and other cognitive impairments, GABAergic, sedative, anticonvulsive and anti-stroke. Phthalides have also been shown to display some progestogenic and cytotoxic effects.

Zebrafish (Danio rerio) is a powerful vertebrate model organism in genetics, drug discovery, developmental biology, and regenerative studies. Zebrafish have become an ideal model for high-throughput screening systems because of their high fecundity, transparency, external development, and short generation times [7][8][9][10]; additionally, ease of housing and maintaining large numbers in captive [11][12][13]. For these reasons, zebrafish have been recognized as a unique model for various human diseases, including Alzheimer’s disease [14][15], diabetes [16], muscular dystrophy [17], and cancer [18]. Zebrafish have many conserved disease proteins with humans. So, the drugs used for humans often have the same effects on these models [19][20]. Even in some of the diseases, zebrafish are considered better models than mice [21]; in this regard, there is a list of small molecules that have been screened in zebrafish and are currently in clinical trials [22].

In contrast to mammalian models, zebrafish can regenerate pancreatic beta cell (pBCs) throughout their entire life [23][24]. Tg(ins:FP-NTR) zebrafish line has been introduced as a model to study pBC regeneration [25][26][27], and the fluorescent protein-nitroreductase (FP-NTR) fusion protein is expressed in pBCs around 24 hours post fertilization (hpf) under the insulin promoter activity [28].

NTR converts metronidazole (MTZ) into a toxin such that exposure of this model to MTZ, results in ablation of NTR-expressing PBCs [29]. Therefore, we generated and established Tg(ins: GFP-NTR) transgenic model in our lab for conducting bioactivity tests of natural products [30].

The promoter activity of fli1, an endothelial-specific transcription factor, was previously used to establish the endothelial-specific transgenic zebrafish lines including Tg(fli1:EGFP) [31].

In this model, the enhanced green fluorescent protein (EGFP) signal is localized to the blood vessels (arteries, veins, and capillaries). So, this model is appropriate for vascular analysis during zebrafish embryonic development [32].

The inhibition of the formation of intersegmental vessels (ISVs) and subintestinal vessels (SIVs) is used as a measure of the potential anti-angiogenesis effects of natural compounds and small molecules; accordingly, Tg(fli1: EGFP) is also an ideal model for cancer research [33][34][35].

The aim of this study was to compare the chemical composition and biological activities/effects of two essential oils (EOs) and hydroalcoholic extracts from K. odoratissima leaves and A. sinensis root. The potential inhibition of angiogenesis and/or induction of pBC regeneration was assessed by a zebrafish-based bioassay system, which can be related to the anti-cancer and/or anti-diabetic activity of the compounds, respectively. To the best of our knowledge, this is the first report on the evaluation and comparison of chemical composition and in vivo bioactivity for K. odoratissima and A. sinensis.

## Materials and Methods

Chemicals and Plant Materials

N-Phenylthiourea (PTU), MTZ, and methylene blue was obtained from Sigma-Aldrich (St. Louis, Missouri, United States). 5′-N-ethylcarboxamidoadenosine (NECA) was purchased from Torcis (Bristol, UK.) and SU5416 was obtained from Abcam (Cambridge, United Kingdom). Ethanol and other chemicals were purchased from Merck (Darmstadt, Germany).

The leaves of K.odoratissima was collected in May 2018 from Bazoft (Chaharmahal & Bakhtiari province, Iran), at an altitude of c. 2500 m. A voucher specimen was deposited in the Herbarium of Medicinal Plants and Drugs Research Institute in Shahid Beheshti University, Tehran (Voucher No: MPH-1414). A. sinensis roots were also collected in Gansu province, China in the autumn of 2018.

Isolation and Analysis of the Essential Oil

One-hundred grams of air-dried leaves of K. odoratissima were powdered and the EOs was isolated by hydrodistillation in a Clevenger-type apparatus for 3 h. The EOs was separated and dried over anhydrous sodium sulfate and kept in the freezer at -20°C until analysis. The same procedure was performed to isolate EOs from the root of A. sinensis.

Preparation of Hydroalcoholic Extracts

The ethanol/water (50%, 100ml) extracts of 10 grams of Kelussia and Angelica were separately prepared by sonication for 30 min. Subsequently, the extract was ﬁltered though Wathman paper (no. 1).

The extracts were concentrated at 40 °C using a rotary evaporator and finally powdered in a freeze drier to remove the residual water. The hydroalcoholic extract of Kelussia leaves (KEL) and Angelica root (AER) were stored at -20 °C until analysis.

GC and GC/MS Analyses and Interpretation of Volatile Oil Components

The isolated EOs were analysed using a GC Agilent 7890B equipped with a flame ionization detector (FID). The HP-5 column length, inner diameter, and film thickness were 30 m, 0.25 mm and 0.25 µm, respectively. The oven temperature was programmed from 60 °C to 280 °C with the ramp of 5 °C/min and held at 280 °C for 2 minutes. Helium was used as the carrier gas at a flow rate of 1.1 mL/min. The detector and injector temperatures were kept at 280 °C and 250 °C, respectively. A Thermoquest-Finnigan gas chromatograph coupled with a trace mass spectrometer (GC/MS) with the same parameter for fused silica column, oven temperature, carrier gas, flow rate, and injector temperature was also used to identify individual peaks. The ionization voltage was set at 70 eV and the interface temperature and ion source were kept at 250 °C and 200 °C, respectively. The identification of the individual components of both EOs was performed by comparing their mass spectra with those of the Adams and Wiley 7.0 internal references mass spectra library and was confirmed by comparing their calculated retention indices (relative to n-alkanes C8-C24) with those reported in literature data [36]. The quantification of the individual component in both samples was performed by relative area percentages using GC-FID.

Zebrafish Models and Maintenance

Tg(ins: GFP-NTR) [28] and Tg(fli1:EGFP) [31] zebrafish strains were used as transgenic models to test the bioactivity of the compounds. Adult zebrafish, both male and female, were mixed and maintained at 28 °C on a 14 h light/10 h dark cycle. Mating was routinely carried out at 28 °C, male and female zebrafish were placed in a breeder basket the night before and embryos were collected in the morning. The embryos were washed and staged as described previously [37]. Approximately 300-400 embryos were generated on average and cultured in E3 media (5 mM NaCl, 0.33 mM MgSO4, 0.33 mM CaCl2, 0.17 mM KCl, and 0.1% methylene blue). Some embryos were raised in the presence of 0.003% PTU, starting from 24 hpf, to prevent pigmentation.

Viability Tests

To test the viability of 12 hpf of Tg(fli1: EGFP) embryos and 4 dpf of Tg(ins: GFP-NTR) larvae, we treated them with five different concentrations of K. odoratissima leaves (KVL), KEL, AER and A. sinensis root (AVR) (500, 125, 31.25, 7.81 and 1.95 µg/ml). At least ten of 4 dpf of Tg(ins: GFP-NTR) larvae were treated for 2 days. Next, the viability of embryos/larvae at each concentration were then reported as percentages and plotted as a viability curve. A similar procedure was used to evaluate the possible pBC regeneration capacities of Kelows extracts (Hexane (KHL), EtOAc (KEtL), MeOH (KML)). The viability test was used to determine the working concentration of each compound. The details are provided in the supplementary data.

Testing Antiangiogenic Activity

Synchronized and healthy Tg (fli1: EGFP) embryos were harvested again at the 6-somite stage (12 hpf). Embryos were placed into 24-well plates, with 10 embryos per well, in 1.5 ml E3 medium and incubated at 28 °C with desired concentrations of each compound that were previously determined by the viability test. Eighteen hours later, using an Olympus SZX16 Fluorescence stereomicroscope equipped with a Canon digital camera, embryos were observed and imaged for blood vessel development, morphological changes, and toxicity. We scored the anti-angiogenic activity of extracts by determining the extent of the ISVs dorsal outgrowth in live transgenic embryos. The mean extent that ISVs extended dorsally from the dorsal aorta/posterior cardinal vein (100, 75, 50, 25, or 0%) was used as a scoring value. The anti-angiogenic compound, SU5416, previously described as the inhibitor of vascular endothelial growth factor (VEGF) receptor [38], was also used as a positive control. In all experiments DMSO treatment (1%) was used as the vehicle, healthy fish were denoted as an untreated group, and NC as the negative control.

Testing PBC Regeneration Activity

Fertilized Tg (ins: GFP-NTR) transgenic zebrafish embryos were collected and incubated at 28 °C for 24 hours. Dead embryos were removed and the E3 medium was replaced with fresh embryo medium containing 0.003% PTU to prevent pigment formation. GFP-positive larvae with green fluorescent pBCs were selected at 3 dpf, and subjected to MTZ treatment at a concentration of 5 mM was performed to induce pBC ablation, so heterozygous transgenic larvae were immersed in MTZ solution for 24 hours. In the following, 10 larvae were placed per well in 24-well plate containing 1.5 ml E3 medium and optimum concentrations for each extract. Forty-eight hours after treatment, live larvae were imaged with a fluorescent stereomicroscope.

PBC regeneration for each compound was evaluated by measuring Pancreatic beta cell arbitrary unit (pBC area AU) of each larva by using ImageJ software (National Institutes of Health (NIH), Bethesda, Maryland, USA). NECA which increases pBC regeneration by activating adenosine G protein-coupled receptor (GPCR) signaling was used as a positive control [39].

Statistical Analysis

Each experiment was carried out at least three times, and all data are presented as mean ± S.D. One-way ANOVA was used to analysis the statistical significance of the data produced from experiments using Graph Pad Prism 6 software (GraphPad Software, Inc., San Diego, CA). P values less than 0.05 were considered significant.

Ethical Statement

All animal procedures were approved by the Royan Institute Ethics Committee with the ethical code IR.ACECR.ROYAN.REC.1398.211 and conducted in full compliance with the guidelines of the Animal Care Committee.

## Results

Chemical Composition of the EOs

Pale yellow colour EOs with especial aroma in 0.03 and 0.04% yield (V/W % relative to dry weight of plant materials) by hydrodistillation process for volatiles of KVL and volatiles of AVR, respectively.

**Figure-1:**
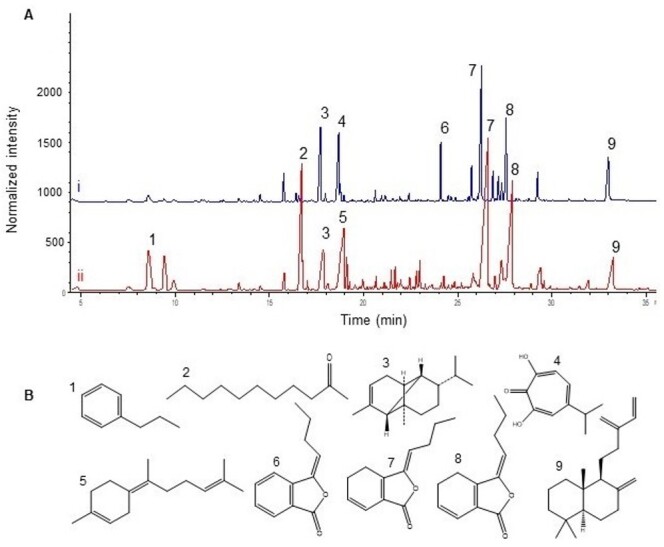
Figure1. **A:** GC chromatograms of essential oils of Kelussia odoratissima (i) and Angelica sinensis (ii); **B:** Structures of some of the main compounds (more than 5%) **1:** n-Propylbenzene, **2:** 2-Undecanone, **3:** α-Copaene, **4:** β-Thujaplicinol, **5:** (Z)-γ-Bisabolene, **6:** (3E)-Butylidene phthalide, **7:** (Z)-Ligustilide, **8:** (E)-Ligustilide, **9:** Sclarene

A total of 52 compounds were identified in both samples, representing 92.7 and 95.8% of the EOs for Kelows and Don qui, respectively. GC chromatograms of AVR and KVL demonstrated that the Z-ligustilide (24.5% in KVL and 19.3 % in AVR) followed by E-ligustilide (14.1% in KVL and 10.1% in AVR) are the major compounds in both samples (Figure-1A section i and ii, respectively).

**Figure-2:**
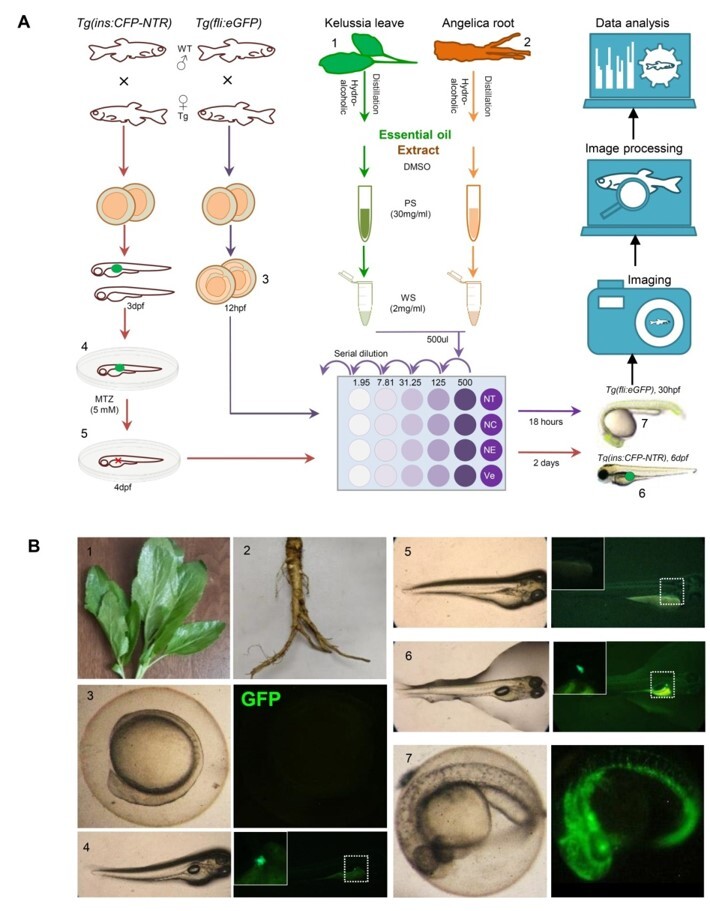
Figure2. **A:** Schematics of zebrafish anti-angiogenesis and pBC regeneration bioassay methods. **B: (1)** Kelussia leave. **(2)** Angelica root. Bright-field and GFP filter view of: **(3)** 12 hpf Tg (fli: EGFP) embryo, **(4)** 3 dpf Tg(ins: GFP-NTR) larvae, **(5)** 4 dpf Tg(ins: GFP-NTR) larvae treated by MTZ, **(6)** 6 dpf Tg(ins: GFP-NTR) larvae, **(7)** 30 hpf Tg(fli: EGFP) embryo. Abbreviations: **WT:** Wild type; **Tg:** transgenic; **dpf:** days post fertilization; **hpf:** hours post fertilization; **MTZ:** metronidazole; **PS:** primary stock; **WS:** working stock; **NT:** Not treated; **NC:** Negative control; **Ve:** Vehicle; **GFP:** Green Fluorescent Protein **NE:** NECA.

**Figure-3:**
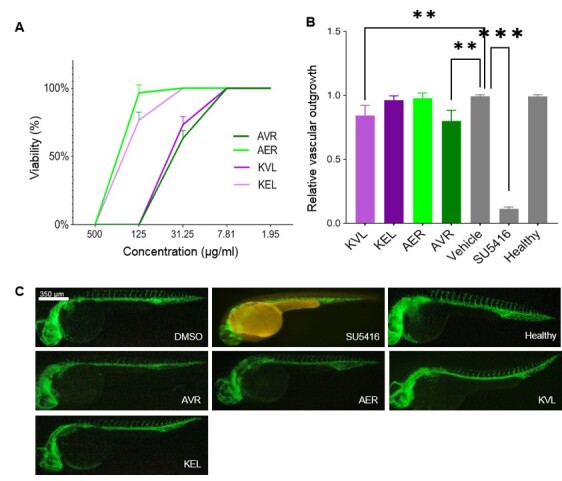
Figure-3. The anti-angiogenesis bioassay in the Tg(fli: EGFP) embryos **A:** Viability rate of zebrafish embryos exposed to different concentrations of extracts and essential oils. **B:** Relative vascular outgrowth in 30 hpf embryos after 18 h exposure to different samples in 7.81 µg/ml concentration (Larvae n:10). **C:** Vascular system of controls and treated zebrafish embryos. All the measurements were taken in triplicates and the values were presented as mean ± SD of three independent experiments, **P<0.01, ***P<0.001. **KVL:** Kelussia volatile oil leaves; **KEL:** Kelussia leaves; **AER:** Angelica root; **AVR:** Angelica volatile oil root.

More specifically, eleven compounds were found to be present in both oils, comprising 51.3% of Kelussia and 61.7% of Angelica EOs, despite the differences in genus, organs, and origin bases. For example, phthalide structures comprise 39.3% of Kelussia and 37.6% of Angelica, including the (Z/E)-ligustilides and (3E/3Z)-butylidene phthalides. The structures of the main components (with a relative percentage of more than 5%) of both EOs included n-Propylbenzene, 2-Undecanone, α-Copaene, β-Thujaplicinol, (Z)-γ-Bisabolene, Sclarene as well as other phthalide structures (Figure-1B). Antiangiogenic Properties of the Compounds The Tg (fli1: EGFP) and Tg(ins: GFP-NTR) transgenic zebrafish embryos were used to evaluate the possible anti-angiogenic and/or pBC regeneration abilities of the compounds derived from KEL and Angelica root (details depicted in Figure-2A and -B). The viability results showed that all compounds, including KVL, KEL, AER, and AVR, were 100% viable for 12 hpf Tg (fli1: EGFP) embryos when administered at final concentrations lower than 7.81 μg/ml (Figure-3A). Results showed that KVL and AVR had significantly (P<0.01) decreased relative vascular outgrowth index compared to the vehicle (1% DMSO) and healthy groups (Figure-3B). However, other compounds including KEL and AER showed no significant effect in this regard. As expected and consistent with previous reports, the positive control, SU5416, decreased the relative vascular outgrowth index significantly compared to the vehicle (1% DMSO) and healthy groups (Figure-3B). To confirm the above results, representative fluorescence microscopy images were presented (Figure-3C).

PBC Regeneration Potentials

**Figure-4:**
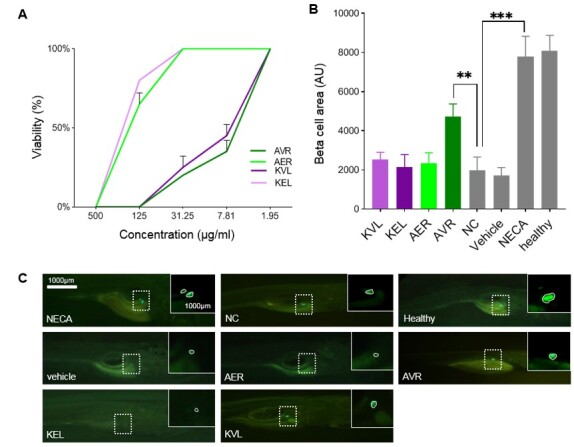
Figure-4. pBC regeneration assay in the Tg(ins: GFP-NTR) larvae. **A:** Viability rate of zebrafish larvae treated with different extractions. **B:** The average pBC area in 6 dpf larvae after regeneration in comparison with the control group. **C:** Fluorescent images of 6 dpf larvae show pBC mass, **P<0.01, ***P<0.001. **KVL:** Kelussia volatile oil leaves; **KEL:** Kelussia leaves; **AER:** Angelica root; **AVR:** Angelica volatile oil root; **NC:** Negative control.

According to the viability test for Tg(ins:GFP-NTR) larvae at 4 dpf, KVL and AVR at a concentration of 1.95 µg/ml, KEL and AER at lower than 31.25 µg/ml showed 100% viability (Figure-4A). The average values of pBC area (AU) for each compound is illustrated in the graph (Figure-4B). According to the results, only the volatile oil from AVR was effective in pBC regeneration (Figure-4C)

and pBC area (AU) significantly (P<0.01) increased in this sample compared to negative and vehicle controls. Other samples, including KVL, KEL and AER were not able to significantly increase the pBC area (AU). NECA as positive control showed the highest regeneration close to the healthy group.

To investigate the bioactivity of Kelussia in more detail, we prepared a series of subsequent fraction extracts; obtained based on the increasing polarities of solvents from KHL, to KEtL and KML. The extraction method for this step was also the same as what we mentioned previously in the Materials and Methods section. Whereas KHL and KEtL induced 100% embryonic viability/survival at

concentrations lower than 7.81 µg/ml, KML was 100% tolerable at the final concentration of 1.95 µg/ml (supplementary Figure-1A). These fractionations showed no significant pBC regenerative capacity (supplementary Figures-1B and -1C).

## Discussion

Regarding the wide range of EOs usability as food ingredients, pharmaceutical agents, and even in the natural pesticide industry, evaluating and discovering their bioactivity potential of them has been of interest throughout history [40][41]. The phytochemical backgrounds of plants along with their bioactivity are also a hallmark of agronomical study and making them an industrial scale crop [42]. Several studies on the chemical and biological properties of Angelica sinensis have brought this plant to prominence, leading to greater consumption and commercialization of this plant, but limited studies on the Kelussia, suggest that its applications were limited to local foods and beverages only. Comparing the aroma of both samples (KVL and AVL), revealed several compounds common to both plants. The analysis of volatile oils also showed a high amount of phthalide structures, especially Z/E-ligustilide in both samples; this may be their most important signature scent. These types of phthalides in both plants had been previously described [43][44] and they are classified as phthalide monomers while they also can be converted to dimer structures. However, according to the molecular weight and volatility, monomers will be isolated only in the EOs. The dimer structures can be obtained in the extracts [45][46][47].

The EOs of Don qui root is higher than the Kelows leaves which is quite reasonable. The root of Kelows also has a higher yield of essential oil, but there is some concern about harvesting the root due to their limited resources [48]. The anti-angiogenesis and pBC regeneration assay was carried out in 12 hpf and 4 dpf zebrafish embryos and larvae, respectively. During these stages, zebrafish rely on yolk sack for feeding [49].

The viability tests for derived compounds from Kelussia and Angelica showed that in different concentrations, embryos for anti-angiogenesis assay showed higher viability compared to larvae in pBC regeneration model. This means that maximum tolerated concentration (MTC), which is usually defined for the zebrafish bioassay systems, is higher for the anti-angiogenic model system, while the used samples in this assay are at the earlier developmental stages compared to the 4 dpf larvae of the pBC regeneration model. This observation could be related to the existence of chorion layers in 12 hpf embryos that reduce compound penetration compared to the 4 dpf larvae. The common part of bioactivity tests in this study belonged to the anti-angiogenic activity of both plant-derived compounds. Previous studies on the effects of Angelica sinensis on angiogenesis have shown variable results and effects.

The proangiogenic activity of Angelica has been reported for in vivo and in vitro assays [50]. A significant anti-angiogeneic activity of this plant has been reported [51]. The most important factor leading to these differences seems to be the difference in extraction methods; Chen et al. used the acetonic extract which could be rich in phthalide structures, while Lam et al. used the commercial extract of Angelica ed to be methanolic extract and contains fewer phthalide structures. On the other hand, the assay method and developmental stage of embryos that were exposed to extract are also crucial in antiangiogenic activity tests. Treatment of small molecule SU5416, as a standard positive control for antiangiogenesis assay, inhibited the ISV outgrowth completely, but after 72 hpf, ISV will be regenerated (data is not shown). Therefore, embryos at 30-48 hpf were chosen for anti-angiogenesis assay, which in this case, the EOs of both samples with a considerable amount of Z/E-ligustilide showed a relative anti-angiogenesis activity.

In higher concentrations of the samples, it causes mainly the developmental delay which is not considered an anti-angiogenesis property, although there is no ISV outgrowth. Figure-3C shows the ISV inhibition by treatment of both EOs of Angelica and Kelussia while there is no significant anti-angiogeneis activity for their hydroalcoholic extracts. By the way, looking for the previous studies on the effect of different extraction solvents of Angelica sinensis, led us to study different extracts of Kelussia and examine its biological activity which shows interesting effects and will be presented in future reports.

The same procedure was carried out on the other hexane, ethyl acetate, and methanol extract of Kelussia on pBC regeneration, which showed no activity; data is presented in supplementary Figure-1S. These extracts were just prepared subsequently according to increasing polarities with the same procedure of extraction described in the Materials and Methods section. In traditional medicine-based studies, most medicinal plants have been tested for their antidiabetic activity in type 2 diabetes [52][53][54].

However, here, we used this transgenic zebrafish model for evaluating the antidiabetic activity of naturally derived compounds for type 1 diabetes and believe that this model can be utilized for screening any synthetic or natural compounds for type 1 diabetes. For the pBC regeneration assay, pBCs were first ablated via cell-specific transgenic expression of NTR. Interestingly, neither KEL nor AER showed a significant effect on pBC regeneration. However, there were some differences in the bioactivity of KVL and AVR, as the regenerated pBC area upon treatment with AVR was significantly higher than those treated with DMSO (Figure-4 B). It suggests that AVR could stimulate proliferation in pBCs of larvae but is not effective as NECA. This difference might be due to the differences between the chemical compositions of both oils. For example, the Angelica oils had 6.7% of (3E)-Butylidene phthalide while Kelussia had 0.2% but for the E-ligustilide it was 14.1% in KVL and 10.1 % in AVR. N-Propylbenzene, 2-Undecanone, (Z)-γ-Bisabolene are the major compounds in KVL while β-Thujaplicinol exclusively was identified in AVR, which could be a possible reason for the different bioactivity on pBC regeneration. There were limited studies on the bioactivity of β-Thujaplicinol that were limited to the inhibition of hepatitis B virus replication [55] and RNase H inhibitor which is a target in the treatment of drug-resistant HIV variants [56].

Other studies have demonstrated the hypoglycemic and hypolipidemic potential of Angelica sciences polysaccharide, in prediabetes and type 2 diabetes mice models wherein the reduction of IL-6 and TNF-α as insulin resistance inflammatory factors and also the simulation of glycogen synthesis and insulin secretion were noted [57][58].

## Conclusion

In conclusion, our study highlights several similarities between K. odoratissima and A. sinensis, in terms of the chemical composition of their volatile compounds and the bioactivities of both volatile oils and total extracts of these plants on angiogenesis and pBC regeneration. Our results could pave the way for new studies into the potential applications of these plants, which are currently limited to folk remedies and local consumption.

In particular, new studies are warranted to test their application in the pharmaceutical and food industries and also for testing their efficacy in inhibiting angiogenesis in different pathologies, such as tumor growth or vascular malformations. By finding the active metabolites and standardization of them in the whole process of plant production, industrialization can also be obtained more feasibly. The other chemical composition of phthalide dimers in the different extracts of Kelussia, similar to Angelica [45][46][47] along with their biological activities is an ongoing project and will be presented in the following publications.

## Acknowledgements

The authors also would like to thank Prof. WeiWei Gao and Dr. Xiaolin Jiao from the Institute of Medicinal Plants and Developments (IMPLAD), Beijing, China, for their kind help and assistance.

The financial support for this project was received from the Royan Institute and Tarbiat Modares University research councils and the Medical Plants and Traditional Medicine Sciences and Technologies Development Headquarters. (grant number 97000190)

## Conflict of Interest

The authors declare that there are not any known competing interests.
